# Outcomes of Traumatic Eyelid Laceration Repairs by Ophthalmology Residents Performed in the Emergency Department

**DOI:** 10.7759/cureus.110056

**Published:** 2026-06-01

**Authors:** Georgia L Schafer, Marina Gad El Sayed, Natalie Chen, Shaili Davuluru, Teresa Chen, Sandy Zhang-Nunes, Jeremiah Tao, Jennifer Hui

**Affiliations:** 1 Ophthalmology, Loma Linda University Medical Center, Loma Linda, USA; 2 Ophthalmology, University of California Riverside School of Medicine, Riverside, USA; 3 Gavin Herbert Eye Institute, University of California Irvine Health, Irvine, USA; 4 Ophthalmology, Keck School of Medicine, University of Southern California, Los Angeles, USA; 5 Ophthalmology/Oculoplastic Surgery, Kaiser Permanente Southern California Permanente Medical Group, Los Angeles, USA; 6 Roski Eye Institute, Keck School Medicine, University of Southern California, Los Angeles, USA; 7 Oculofacial Plastic Surgery, Keck School Medicine, University of Southern California, Los Angeles, USA; 8 Oculofacial Plastic Surgery, University of California Irvine Health, Irvine, USA; 9 Ophthalmology/Oculofacial Plastic Surgery, Loma Linda University Medical Center, Loma Linda, USA

**Keywords:** eyelid laceration, eyelid repair, ocular trauma, oculoplastic procedures, resident education, trauma

## Abstract

Introductory statement

Eyelid lacerations are a common problem in the emergency department (ED) that ophthalmology residents manage. This study aims to evaluate the outcomes of eyelid lacerations repaired by ophthalmology residents.

Methods

A multicenter retrospective study was performed on 287 patients who presented to the ED with eyelid lacerations that were repaired by an ophthalmology resident at one of the four Southern California institutions (Loma Linda University (LLU), Riverside University Health System (RUHS), University of California Irvine (UCI), and Los Angeles General Medical Center (LAGMC)).

Results

Approximately half of the patients (134; 47%) were lost to follow-up. Of the patients with follow-up, there was no statistically significant difference in re-operation rate for lacerations repaired by PGY-2 residents with supervision (7/21; 35%) and without supervision (6/32; 19%). When a PGY-2 performed the procedure as the primary surgeon solo in the emergency room, the complication rate was 41% (13/32). When the PGY-2 had the assistance of a senior resident, fellow, or attending, the complication rate dropped to 29% (6/21). The complication rate for an upper-level resident (PGY-3 or PGY-4) performing the repair in the ED was 23% (5/22) and 17% (1/6) if supervised by a fellow or attending (p=1.00). The most common complication was eyelid notching (10/153; 6.5%), followed by epiphora (9/153; 5.9%) and blepharoptosis (7/153; 4.6%).

Conclusions

Approximately half of the patients who underwent eyelid laceration repair in the ED were lost to follow-up. Of the patients who followed up and are included in this data set, the overall complication rate was similar to previously recorded complication rates recorded in the literature. Re-operation rates were not significantly different when the PGY-2 resident operated alone compared to repair with supervision. Residents were appropriately calling for supervision when dealing with a complicated laceration.

## Introduction

Eyelid lacerations are a common ophthalmologic pathology in emergency rooms and may pose significant challenges [1]. They are one of the most common ocular trauma consults in the emergency department (ED), and the incidence ranges from 243 to 185 per million patients annually [2]. The intricate anatomy of the eyelid, coupled with the potential for functional and aesthetic complications, necessitates a thorough understanding of repair techniques, potential pitfalls, and strategies for optimal outcomes [3]. While relatively uncommon, complications of eyelid laceration repairs do occur and can include lid notching, ectropion, epiphora, hypertrophic scars, ptosis, tearing, and lagophthalmos [4]. In a large retrospective study, Doğan et al. found that eyelid laceration repairs had a 96.6% anatomical success rate and 86% functional success rate [5]. 

Complications can be due to delayed closure, improper tissue approximation, the mechanism of injury, the location and extent of the laceration, or the inherent complexity of the injury [6,7]. Deep lacerations, especially those involving the lacrimal system, pose a greater challenge for repair and are associated with lower anatomical and functional success rates [8]. The time to laceration repair is also important, with current guidelines recommending repair of canalicular eyelid lacerations in under 48 hours [9]. The skill and experience of the surgeon also play a pivotal role in mitigating complications. Bedside repairs in the ED obviate many risks and costs associated with surgery in the operating room (OR). These include timely repair as well as the need for anesthesia and other necessary OR staff. Because of this, at many academic institutions, ophthalmology residents perform bedside eyelid laceration repairs in the ED. These surgeries often afford the first primary surgeon experience to ophthalmology residents. The Accreditation Council for Graduate Medical Education (ACGME) requires ophthalmology residents to perform three primary eyelid laceration repairs to fulfill graduation requirements. This is a very low number to become proficient at this procedure; however, depending on how much trauma different programs see, the opportunity residents have to repair eyelid lacerations varies greatly. According to Abousy et al., the average ophthalmology resident documents that they repair 9.58 lacerations, while the minimum documented was only one repair, and the maximum was 52 repairs. They did not find any significant correlation between programs that had oculoplastic fellows available compared to those that did not [10]. This study seeks to describe the outcomes of eyelid laceration repairs performed bedside in the ED by ophthalmology residents.

## Materials and methods

After obtaining approval from the Institutional Review Board of Loma Linda University Health (approval number: 5240435), a multicenter retrospective study was conducted at Loma Linda University (LLU) in Loma Linda, California; Riverside University Health System (RUHS) in Moreno Valley, California; University of California Irvine (UCI) in Irvine, California; and Los Angeles General Medical Center (LAGMC) in Los Angeles, California. Individual consent was waived at all sites. 

**Figure 1:**
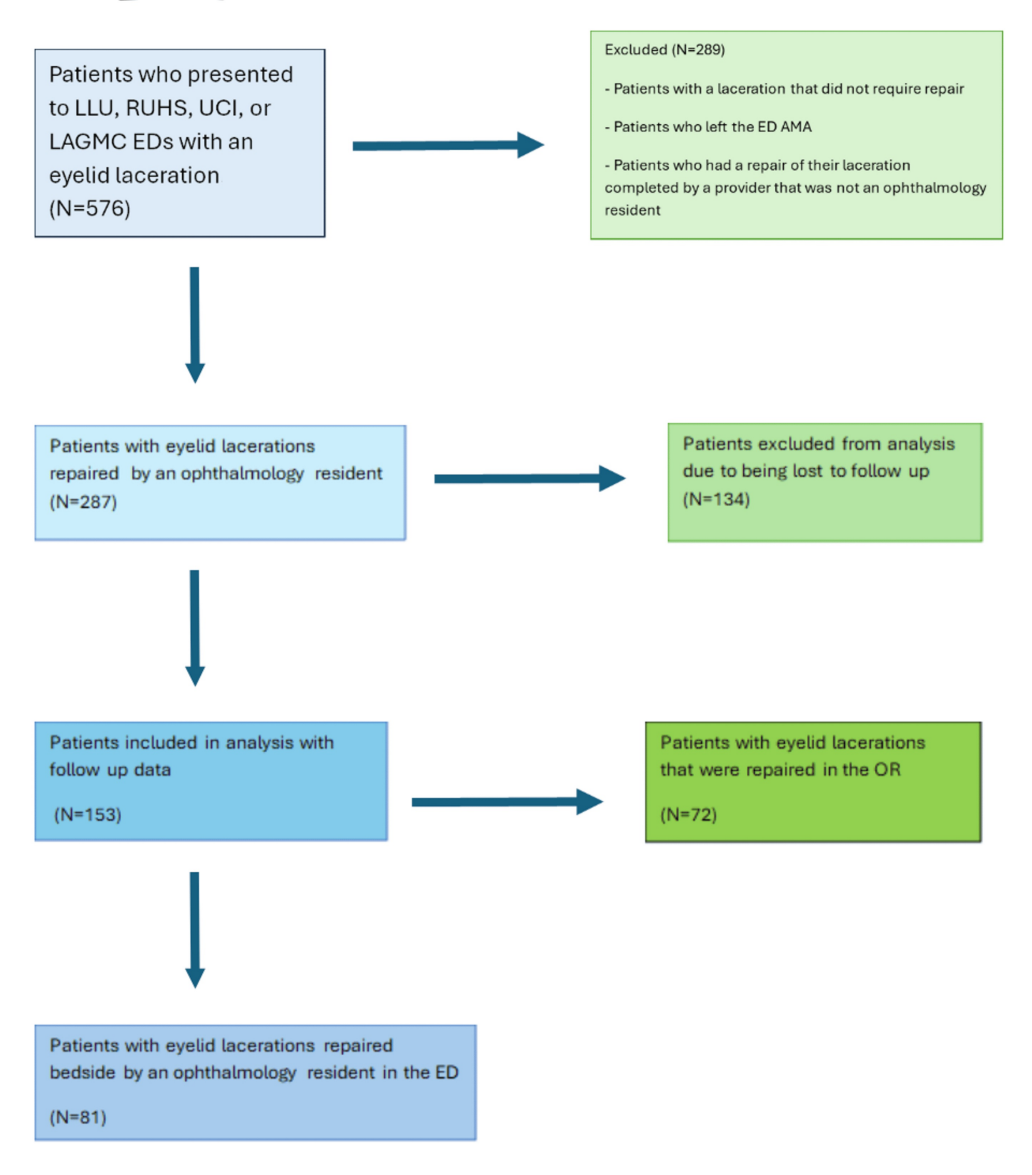
Cohort flow study LLU: Loma Linda University; RUHS: Riverside University Health System; UCI: University of California Irvine; LAGMC: Los Angeles General Medical Center; ED: emergency department; AMA: against medical advice; OR: operating room

Inclusion criteria were patients presenting to the ED with an eyelid laceration that was repaired by an ophthalmology resident. Exclusion criteria included patients who had lacerations that were mild enough to not require repair, patients who left against medical advice (AMA), patients who did not have a documented eyelid laceration repair procedure or documented eyelid laceration on exam, and patients who had a laceration that was repaired by a provider who was not an ophthalmology resident. Figure 1 outlines the inclusion and exclusion criteria in a cohort flowchart.

Insurance, patient age and gender, cause of laceration, description of laceration and grading of difficulty, location of repair (i.e., ED vs. OR), level of training of the resident or fellow performing the repair and level of training of their supervisor, other concomitant injuries (i.e., globe rupture, orbital fracture, etc.), and follow-up were recorded. In patients who did return to the clinic, the duration of follow-up, the number of further surgeries that were required relating to the eyelid repair and when they were performed, and other complications that resulted from the repair were recorded. The study was Health Insurance Portability and Accountability Act (HIPAA)-compliant and followed the Declaration of Helsinki principles; no protected health information was shared with other sites. 

At LLU and RUHS, the data were collected separately using Epic SlicerDicer from ED encounters between 2015 and 2024. At LAGMC, patients of the LAGMC oculoplastic clinic with a history of eyelid laceration were identified between May 2022 and December 2024. At UCI, patients who underwent eyelid laceration repair from January 2020 to October 2024 were identified using Current Procedural Terminology (CPT) billing codes 67935 and 68700. 

The lacerations were graded on the following 1-5 scale. Grade 5 was a simple non-margin involving laceration. Grade 4 was a complex non-margin involving laceration, for example, a stellate laceration or a deeper laceration involving musculature. Grade 3 was a margin involving laceration. Grade 2 was a simple canalicular or tendon involving laceration. Grade 1 was a complex canalicular, tendon, or levator involving laceration requiring extensive repair. If the patient had multiple lacerations, they were given the grade of the most complex laceration.

Statistical analysis 

Descriptive statistics are reported as mean±standard deviation with the minimum and maximum values for continuous variables and as frequencies with percentages for categorical variables. 

Pearson's chi-squared test and Fisher's exact test were used to assess the associations between procedures performed by second-year residents, either alone or with supervision, and the level of difficulty as well as post-procedure complications and outcomes. 

Statistical analyses were conducted using IBM SPSS Statistics for Windows, Version 29.0 (IBM Corp., Armonk, New York, United States). Alpha was set at a significance level of 0.05.

## Results

Data were analyzed from 287 patients who presented to the ED with eyelid lacerations repaired by an ophthalmology resident, of which 136 (47%) were from LLU, 65 (23%) were from RUHS, 54 (19%) were from UCI, and 32 (11%) were from LAGMC. Of these patients, 187 (65%) underwent bedside repair of their laceration in the ED, while the rest were taken to the OR for repair.

The patients who were lost to follow-up were not included in the analysis of re-operation rates or complications. Of the 153 patients (53%) who presented for follow-up appointments, 81 patients (53%) had lacerations that were repaired bedside in the ED. Thirty-two patients (40%) had lacerations that were repaired by a PGY-2 resident as the primary physician alone, 21 lacerations (26%) were repaired by a PGY-2 resident with the assistance of a senior resident (8; 38%), fellow (5; 24%), or attending (8; 38%), 22 lacerations (27%) were repaired by a senior resident (PGY-3 or PGY-4) solo, six lacerations (7%) were repaired by a senior resident with the supervision of a fellow (4; 67%) or attending (2; 33%), and the remaining were repaired by a fellow or attending.

**Figure 2:**
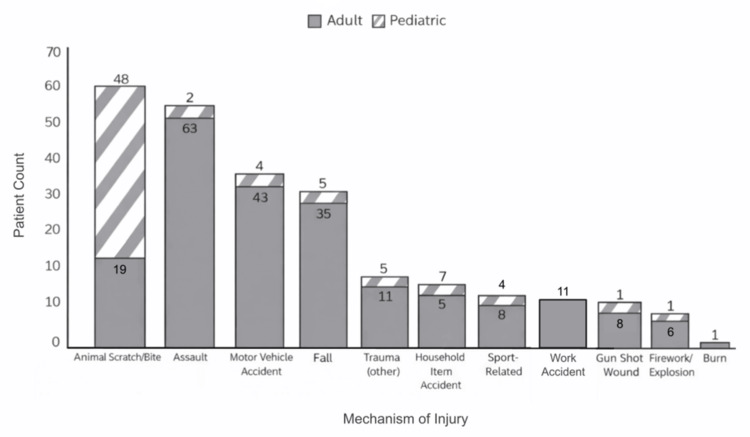
Pediatric and adult causes of eyelid lacerations that presented to the emergency room

The mechanism of eyelid lacerations was varied and different for the pediatric (<19 years old) versus the adult patient population. In pediatric patients, most eyelid lacerations were animal-related injuries, commonly dog bites. In the adult population, the most common cause was assault. A graph of the causes of eyelid lacerations divided by pediatric and adult patients is shown in Figure 2.

**Figure 3:**
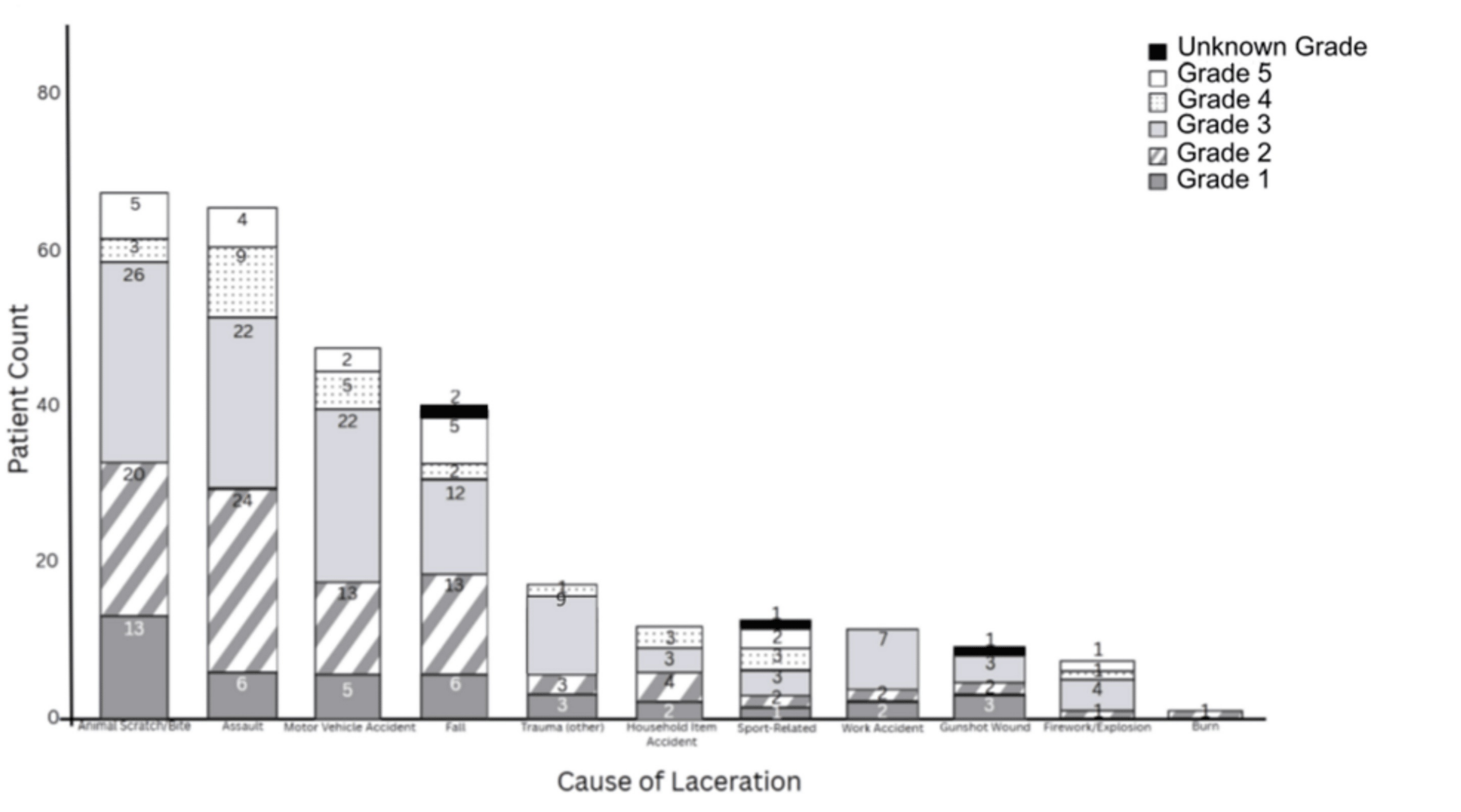
Grade of eyelid laceration compared to cause of eyelid laceration

The size and severity of the lacerations also varied based on the cause of the laceration. The grade of laceration complexity compared to the cause of the laceration is shown in Figure 3. Lacerations that were caused by animal bites or scratches were often more complex and involved the canalicular system, with 49% (N=33) of the injuries being grade 1 or 2.

**Figure 4:**
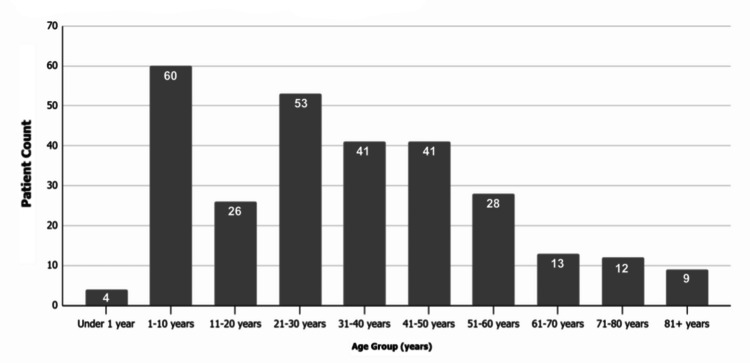
Age of patients that presented to the emergency room with eyelid lacerations

The age of patients who presented with eyelid lacerations was dispersed over all age groups, with the most common presentation being within the first decade of life. Of those who required an eyelid laceration repair, 27% (N=77) were pediatric patients (<19 years old), while 73% (N=210) were adult patients. Most patients with this injury were male (201; 70%). A graph of the age distribution of the patients included in this study is shown in Figure 4.

The total complication rate of laceration repair by residents in the ED was 31% (25/81): 33% (18/54) without supervision versus 26% (7/27) with supervision (p=0.5). There were a variety of complications of eyelid laceration repair. The most common complication overall was eyelid notching (10; 6.5%), followed by epiphora (9; 5.9%), ptosis (7; 4.6%), cicatricial ectropion (5; 3.3%), and corneal irritation due to a suture (5; 3.3%).

When a PGY-2 performed the procedure as the primary surgeon solo in the emergency room, the complication rate was 41% (13/32). When the first-year resident had assistance from a senior resident, fellow, or attending while performing the repair, the complication rate dropped to 29% (6/21) (p=0.37). Of these cases in which the PGY-2 resident had backup, it was a senior resident 38% of the time (complications: 2/8), a fellow in 24% (complications: 2/5), and an attending in 38% (complications: 2/8). When a senior resident performed the repair solo in the ED, the complication rate was 23% (5/22). When a senior resident performed eyelid laceration repair in the ED with supervision of a fellow (67%) or attending (33%), the complication rate was 16.7% (1/6) (p=1.00). Complications were only reported if the complication was related to the eyelid laceration repair, for example, globe trauma complications were not included.

For a PGY-2 resident performing the eyelid laceration repair solo in the ED, six out of 32 patients (19%) required further surgical repair. When there was a senior resident, fellow, or attending present, further surgery was needed in seven out of 21 patients (33%). The difference was not statistically significant (p=0.719). When a PGY-3 or a PGY-4 performed the repair solo, three out of 22 (14%) patients required further surgery. When a fellow or attending was assisting the PGY-3 or PGY-4 in the ED, no patients (0/6) required further surgery (p=1).

The trainee surgeon was unable to complete the repair or unable to place a canalicular stent in a few cases. Four repairs where a PGY-2 was the primary surgeon were unable to be adequately completed in the ED and required continuation in the OR setting with supervision by an attending. For 15 patients who had a canalicular involving laceration, a canalicular stent was unable to be placed when it was indicated; it was indicated in 41 patients in total (37%). When the PGY-2 was performing the repair alone in the ED, they were unable to place a stent in nine out of 12 canalicular lacerations (75%). When they had the assistance of a senior resident, fellow, or attending, they were unable to place the stent in three out of 16 canalicular lacerations (19%) (p<0.003). When a PGY-3 or a PGY-4 was performing a canalicular repair solo, they were unable to place the stent in three out of seven times (43%), while when they had the assistance of a fellow or attending, they were always able to place the stent (unable: 0/6) (p=0.192).

**Figure 5:**
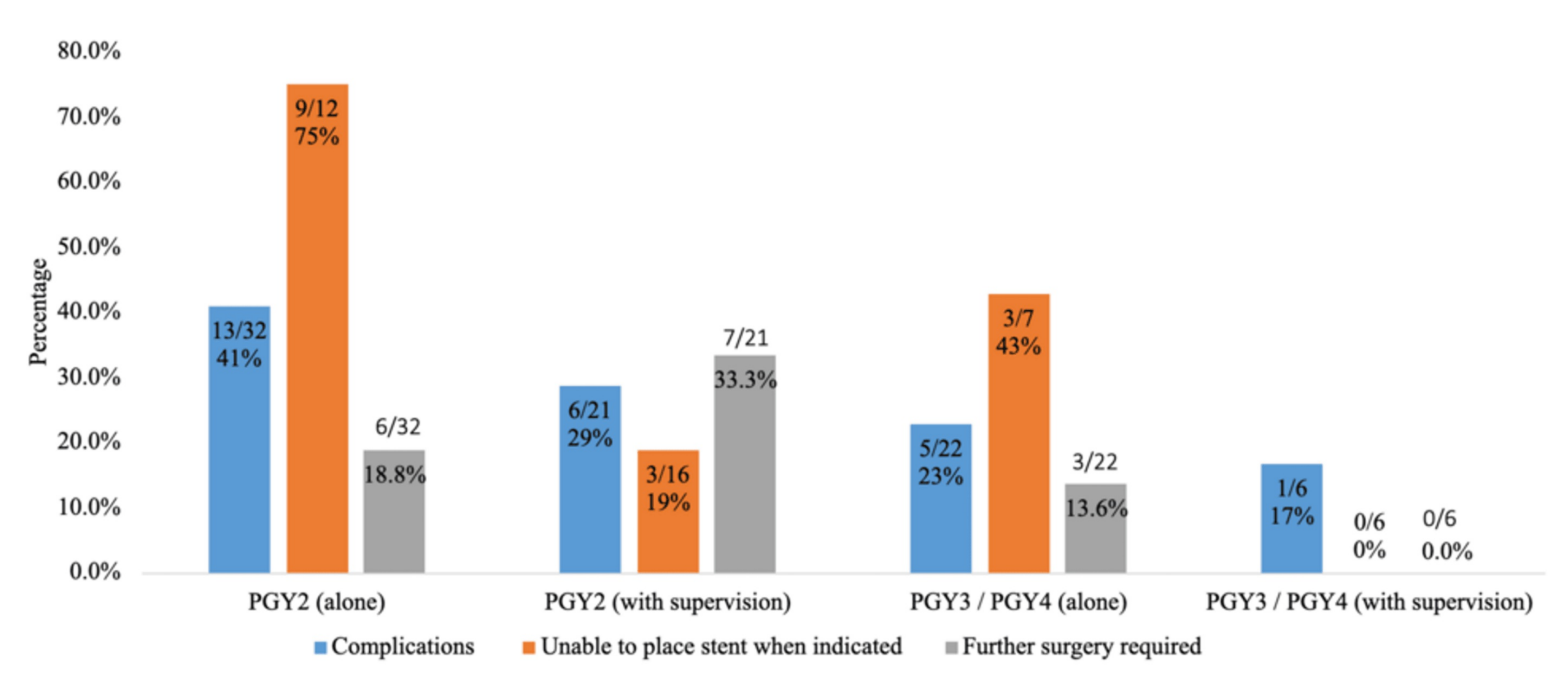
Complication rate, rate of inability to place stent, and rate of further surgery required with regard to the level of training of resident and supervision

Figure 5 shows the rate of complications, re-operation rates, and repairs where a canalicular stent was unable to be placed when indicated in comparison to the different postgraduate training levels of the residents who were performing the repair in the emergency room. Overall, there were a higher complication rate and a need for more surgery following eyelid laceration repair between first-year residents performing the repair solo or when they had supervision; however, it was not statistically significant.

There was a significant difference (p<0.001) between the difficulty of the patients' lacerations for the repairs that took place with a supervising fellow or physician present versus when the resident was performing the repair solo, as shown in Table 1. For the more difficult lacerations that were grade 1 or 2, a senior resident, fellow, or attending was called for supervision 77% (N=96) of the time, while for simple lacerations (grade 4 or 5), the resident only called in supervision 29% (N=13) of the time.

**Table 1:** Laceration difficulty for residents operating alone versus when they were supervised by a fellow or attending *: statistically significant

		PGY-3/PGY-4 alone		PGY-3/PGY-4 with supervision		P-value
		n	%	n	%	
Difficulty of laceration	1-2	11	22	75	83	<0.001*
	3	26	52	12	13	
	4-5	13	26	3	3	

​Overall, 134 patients (47%) were lost to follow-up; of these, 106 (57%) patients underwent repair in the ED. These patients were not included in the re-operation rate or complications analysis since further specifics of their outcome remained unknown. There was no significant difference between the patients who had public and private insurance attending the follow-up appointments.

## Discussion

According to these data, there was a higher rate of complications, aborted repairs, or inability to place a canalicular stent when junior residents were attempting to perform the repair alone. However, the rate of complications did not reach statistical significance. This was likely due to the fact that residents were appropriately escalating the level of care and calling in backup senior residents, fellows, or attendings when needed. There was a statistically significant difference in the difficulty of the lacerations that were repaired by residents alone versus those that were taken to the OR and repaired with the help of attendings or fellows. This shows that residents were appropriately trained to know their limits and understand what was a good procedure to do solo in the ER versus the procedures that required further supervision.

The complication rate noted for a PGY-2 resident repairing an eyelid laceration solo was also higher than complication rates previously reported in the literature, which ranged between 6% and 24% (the large range is due to authors' varying definitions of a complication) [11,12]. However, the complication rate of a senior resident performing the repair solo, or that of a PGY-2 performing the repair with supervision, corroborates rates previously reported in the literature. Most of the complications were not significant or vision-threatening. Many of these patients either didn't require or elected not to undergo further repair. The re-operation rate when a first-year resident was performing the repair as the primary surgeon versus when they had a supervising ophthalmology physician was not significantly different.

The finding of increased complications with procedures performed by novice surgeons is not unexpected. Hashemi et al. found that new PGY-2 residents had a lower rate of diagnostic accuracy on call; however, their diagnostic accuracy rapidly improved throughout their PGY-2 [13]. Similar studies have previously evaluated the complication rates following cataract surgery when residents were operating. In a study by Xiao et al., they found that visual outcomes were similar between resident and attending cases [14]. However, Oetting found that there was a significantly higher rate of intraoperative complications during a resident's first 80 cataract cases [15]. This study corroborates prior literature suggesting experience may correlate with quality of eyelid trauma management [16].

Mono-canalicular stents, such as the Mini-Monoka stent, can be performed bedside in the ED. Properly seating the tube at the eyelid margin as well as locating and intubating the distal end of the canaliculus may be associated with a learning curve. There was a statistically significant decrease in the ability of the resident to successfully place the stent when they were operating solo.

Patient education and follow-up have been associated with lowering the risk of complications [17]. In this study, a large percentage of the patients included were lost to follow-up. Amat et al. performed a large retrospective cohort study at an academic center to evaluate factors that led to poor patient follow-up and found that patients with public insurance were at increased risk of poor follow-up. They also found that patients were at higher risk of being lost to follow-up when they were younger than 40 years old, their physician provider was a trainee, English was not their first language, they primarily identified as non-White, and a myriad of other reasons [18]. The majority of patients included in this study had public or no insurance and were under the age of 40. All of the patients included in the study had a primary provider who was a trainee. Higher social vulnerability index factors likely contributed to the high rate of patients who were lost to follow-up.

There was heterogeneity between the different sites in practices and protocols for managing eyelid laceration repairs in the ED versus the OR, as well as varying protocols in place for supervision. There was a large percentage of repairs that were performed in the OR, which were excluded from the analysis of complication and re-operation rate in this study, primarily because the repairs that were taken to the OR were significantly more complicated and therefore would bias the results.

The complications seen after eyelid laceration repairs by PGY-2 residents should be balanced against the educational aspects of these experiences. Commonly, eyelid lacerations in the ED offer unique, early-in-training autonomous surgeon experiences. Moreover, bedside repair may be more efficient in terms of timeliness of treatment and cost, including no need for OR time and personnel. In fact, the cost of repairing lacerations in the emergency room can be less than two-thirds the cost of a repair in a similar laceration in the OR [19].

Limitations of the study

One of the main limitations of this study is the sample size. Due to many patients being lost to follow-up and the need to stratify laceration repairs by the year of the resident performing the repair and the difficulty of the laceration, the different re-operation rates were not statistically significant. Due to the nature of care given in the ED, many patients never attended their follow-up appointment. This could lead one to the conclusion that these patients did not have complications and therefore required no further care. However, the more likely explanation is that many of these patients lacked the means to attend follow-up appointments or did not have insurance that covered them at the desired site. While there should be a global period where the patient's postoperative visits are covered after they undergo a procedure, many procedures performed by residents are not billed. Therefore, the patient's postoperative visits are not covered which could correlate to Amat et al.'s finding of higher rates of patients who are lost to follow-up when a resident is the patient's primary provider [18]. Other limitations of the study include that the data collection method varied slightly between each site. At LLU and RUHS, data were collected directly through ED encounters, while at USC, data were collected through follow-up visits which excluded some patients who did not attend their follow-up. At UCI, data were collected through billing data; therefore, some patients were excluded when the procedure was not billed. There was a significant difference in the difficulty of the lacerations repaired by a resident solo and those repaired with supervision that was not accounted for. Encouragingly, residents requested supervision or backup with more complicated lacerations. It would be helpful to add additional sites in the future from different geographic areas of the United States to help gain a broader picture of outcomes of resident repairs of eyelid lacerations and increase the generalizability of this study.

## Conclusions

In this multicenter retrospective study, eyelid repairs performed by residents in the ED had a moderate and anticipated amount of complications. Ophthalmology residency can be very challenging. Most residency programs are structured around an apprenticeship model where incoming residents start by watching, then they proceed to performing a procedure or seeing a patient with direct supervision, next they start performing clinical or procedural tasks independently, and then finally they move onward to a place where they are teaching the residents coming up behind them. This has been the structure of medical training for over a century. Residents are a critical part of the healthcare team, and their education is crucial to producing the next generation of physicians. This study found that ophthalmology residents are providing quality care to patients who come to the ED with eyelid lacerations. They are appropriately calling for help, and the overall complication rates are comparable to previously described complication rates. It is so important for junior residents to have sound decision-making and know when to call a senior resident or attending to assist with a complicated pathology. The educational benefits of first autonomous primary surgery experiences should be balanced against the possibility of less optimal outcomes of eyelid laceration repair by novice surgeons in training. Further study may be warranted to assess the financial implications of the model of early trainee performing bedside ED repairs.
